# Supplementary material for: Orientation‐independent quantification of macromolecular proton fraction in tissues with suppression of residual dipolar coupling
Source: NMR Biomed. 2024 Nov 13;38(1):e5293. doi: 10.1002/nbm.5293 (PMC11602536; doi:10.1002/nbm.5293)
Supplement: Supplementary file 1 — Figure S1. Maps of relaxation rates and MPF of knee specimen S2 at different orientations. Top to the bottom: maps of R2, on‐resonance R1ρ at FSL 500 Hz, R1, Rmpfsl, MPFSL, and MPFST. Left to right: maps at orientations of 0°, 15°, 30°, 45°, 60°, 75°, and 90° with respect to B 0. PDW images are included as anatomical reference images. Figure S2. (a) Illustration of two ROIs in knee specimen S2. (b) Anisotropy rate of relaxation rate maps (R2, R1ρ, R1, and Rmpfsl) and MPF maps (MPFSL and MPFST) in ROI1 and ROI2. (c‐h) Mean ± standard deviation of relaxation rates in ROI1 and ROI2, as shown in (a), were calculated at different angle orientations (c) R2, (d) R1ρ, (e) R1, (f) Rmpfsl, (g) MPFSL, and (h) MPFST. Figure S3. Maps of relaxation rates and MPF of knee specimen S3 at different orientations. Top to the bottom: maps of R2, on‐resonance R1ρ at FSL 500 Hz, R1, Rmpfsl, MPFSL, and MPFST. Left to right: maps at orientations of 0°, 15°, 30°, 45°, 60°, 75°, and 90° with respect to B 0. PDW images are included as anatomical reference images. Figure S4. (a) Illustration of two ROIs in knee specimen S3. (b) Anisotropy rate of relaxation rate maps (R2, R1ρ, R1, and Rmpfsl) and MPF maps (MPFSL and MPFST) in ROI1 and ROI2. (c‐h) Mean ± standard deviation of relaxation rates in ROI1 and ROI2, as shown in (a), were calculated at different angle orientations (c) R2, (d) R1ρ, (e) R1, (f) Rmpfsl, (g) MPFSL, and (h) MPFST. Figure S5. Maps of relaxation rates and MPF of knee specimen S4 at different orientations. Top to the bottom: maps of R2, on‐resonance R1ρ at FSL 500 Hz, R1, Rmpfsl, MPFSL, and MPFST. Left to right: maps at orientations of 0°, 15°, 30°, 45°, 60°, 75°, and 90° with respect to B 0. PDW images are included as anatomical reference images. Figure S6. (a) Illustration of two ROIs in knee specimen S4. (b) Anisotropy rate of relaxation rate maps (R2, R1ρ, R1, and Rmpfsl) and MPF maps (MPFSL and MPFST) in ROI1 and ROI2. (c‐h) Mean ± standard deviation of relaxation rates i [file NBM-38-e5293-s001.docx]

**Supplementary Information for**

**Orientation independent quantification of macromolecular proton fraction in tissues with suppression of residual dipolar coupling.**

**This PDF file includes:**

Supporting text

Figures S1 to S12

Supplementary Information Text

The Supplementary information includes Figure SI1-SI12, which displays the experimental result of from specimen 2 (S2) to specimen 7 (S7).


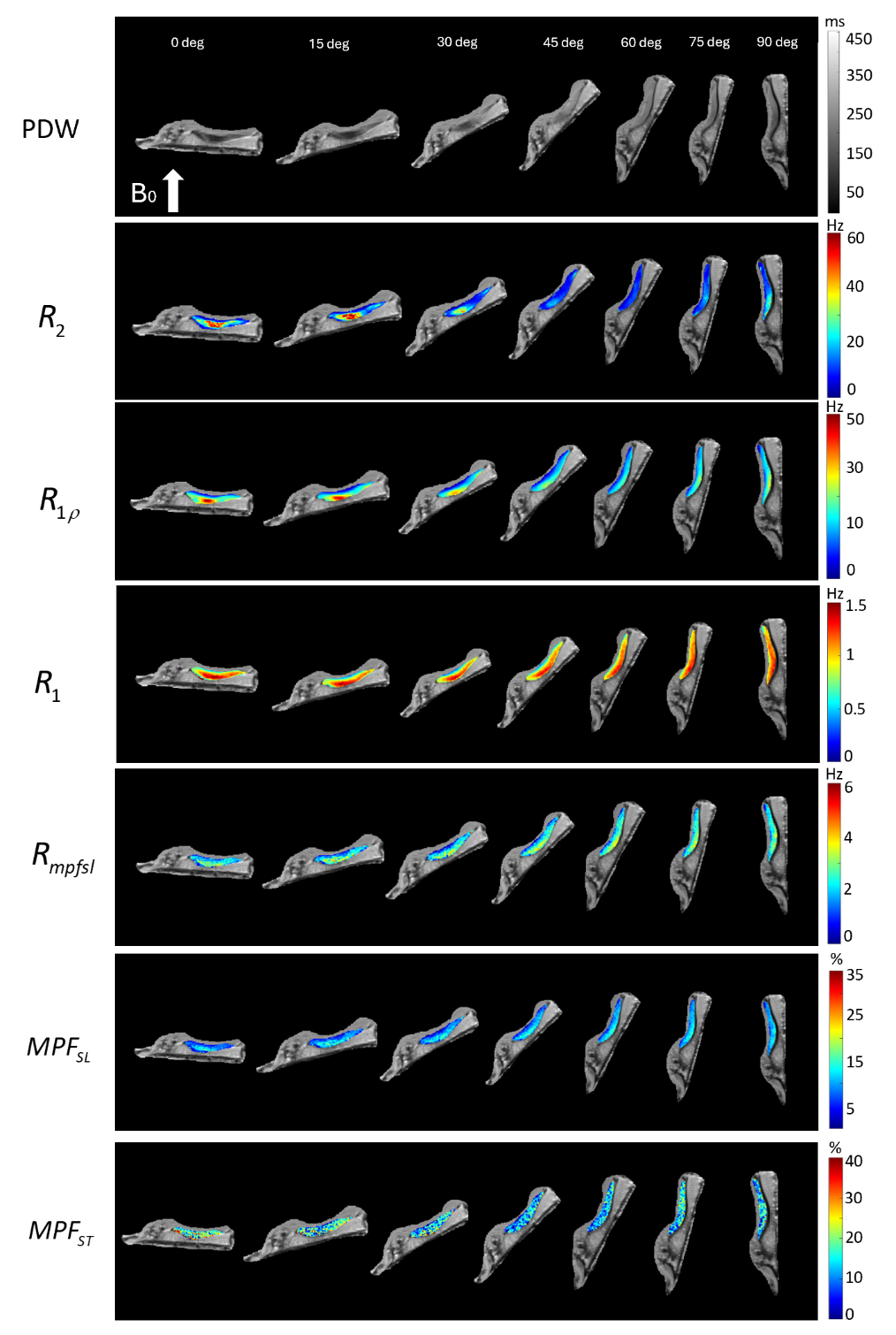


Figure. S1. Maps of relaxation rates and MPF of knee specimen S2 at different orientations. Top to the bottom: maps of $R_{2}$, on-resonance $R_{\text{1ρ}}$ at FSL 500 Hz, $R_{1}$, $R_{mpfsl}$,, $MPF_{SL}$, and $MPF_{ST}$. Left to right: maps at orientations of 0°, 15°, 30°, 45°, 60°, 75°, and 90° with respect to *B*_0_. PDW images are included as anatomical reference images.


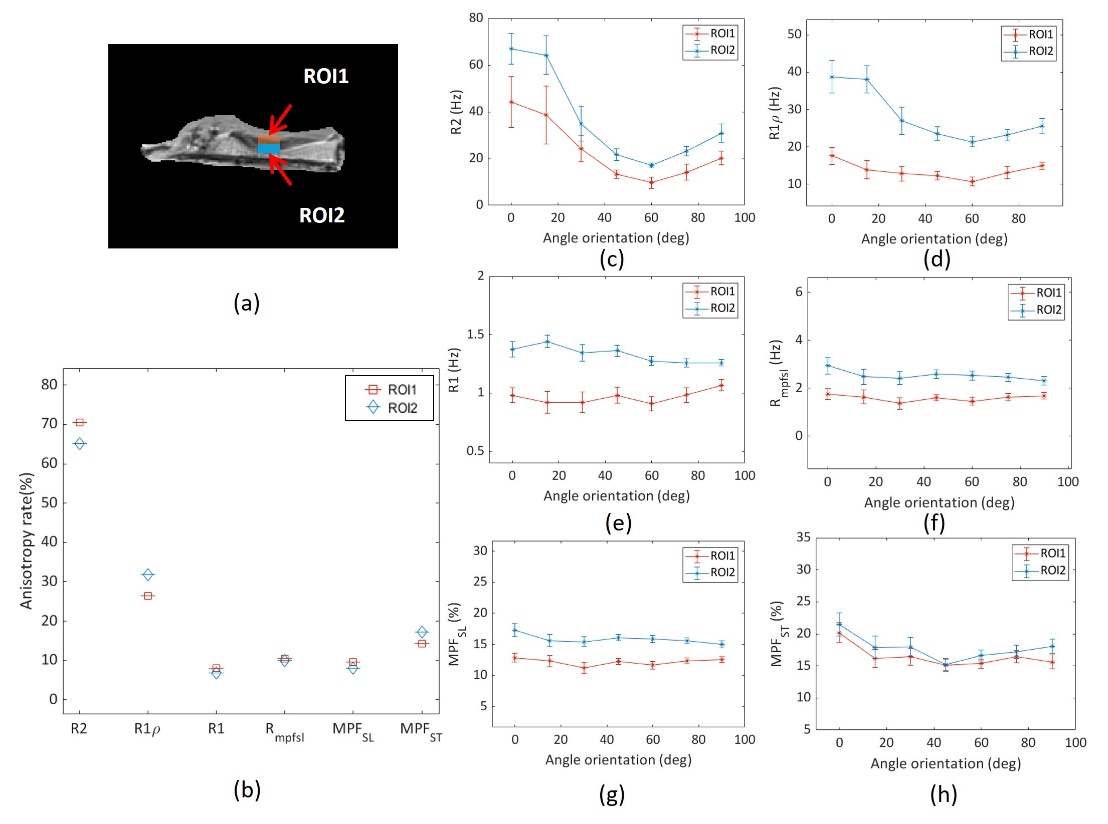


Figure. S2. (a) Illustration of two ROIs in knee specimen S2. (b) Anisotropy rate of relaxation time maps ($R_{2}$, $R_{\text{1ρ}}$, $R_{1}$, and $R_{mpfsl}$) and MPF maps ($MPF_{SL}$ and $MPF_{ST}$) in ROI1 and ROI2. (c-h) Mean ± standard deviation of relaxation rates in ROI1 and ROI2, as shown in (a), were calculated at different angle orientations (c) $R_{2}$, (d) $R_{\text{1ρ}}$, (e) $R_{1}$, (f) $R_{mpfsl}$, (g) $MPF_{SL}$, and (h) $MPF_{ST}$.


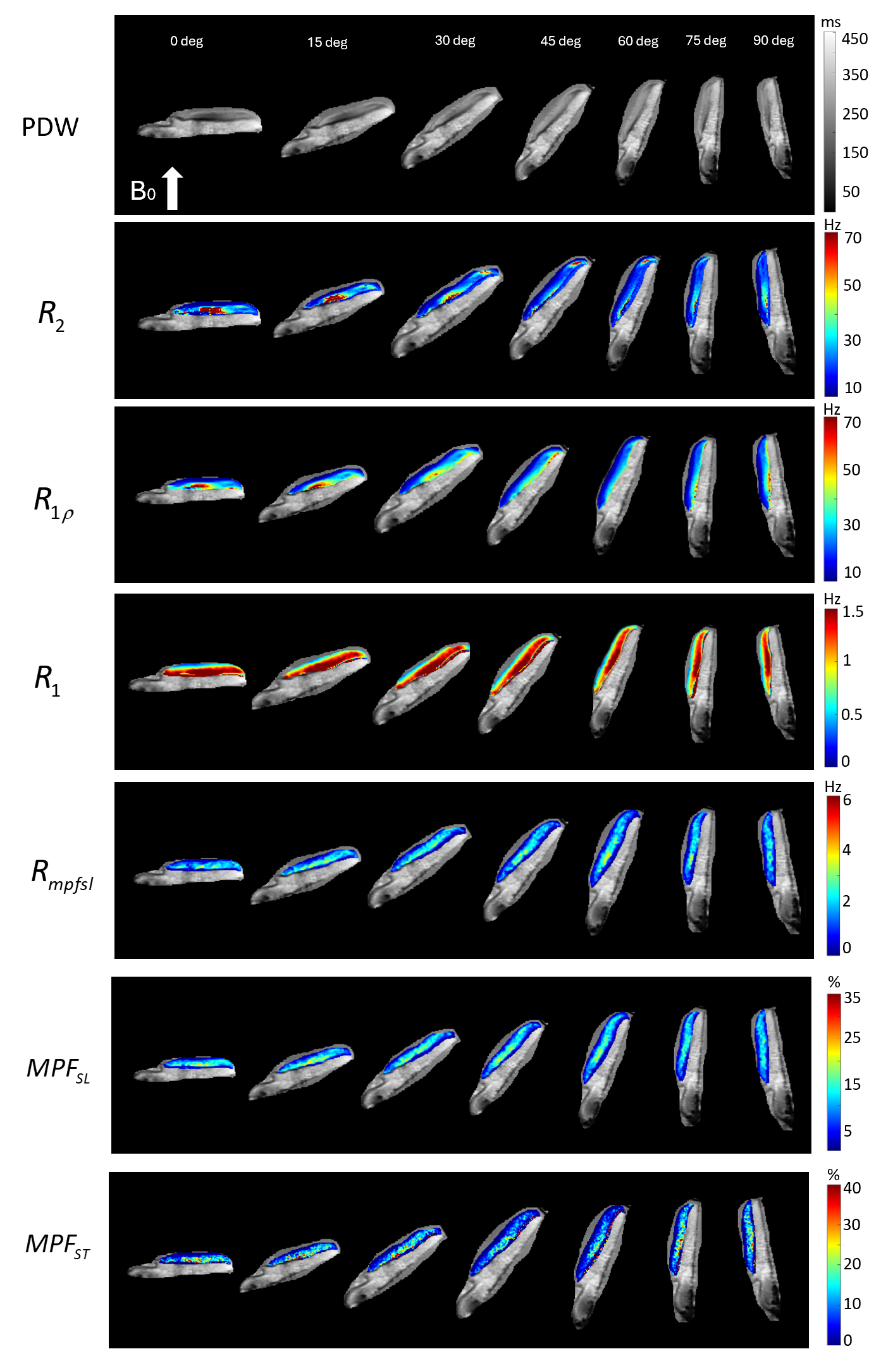


Figure. S3. Maps of relaxation rates and MPF of knee specimen S3 at different orientations. Top to the bottom: maps of $R_{2}$, on-resonance $R_{\text{1ρ}}$ at FSL 500 Hz, $R_{1}$, $R_{mpfsl}$, $MPF_{SL}$, and $MPF_{ST}$. Left to right: maps at orientations of 0°, 15°, 30°, 45°, 60°, 75°, and 90° with respect to *B*_0_. PDW images are included as anatomical reference images.


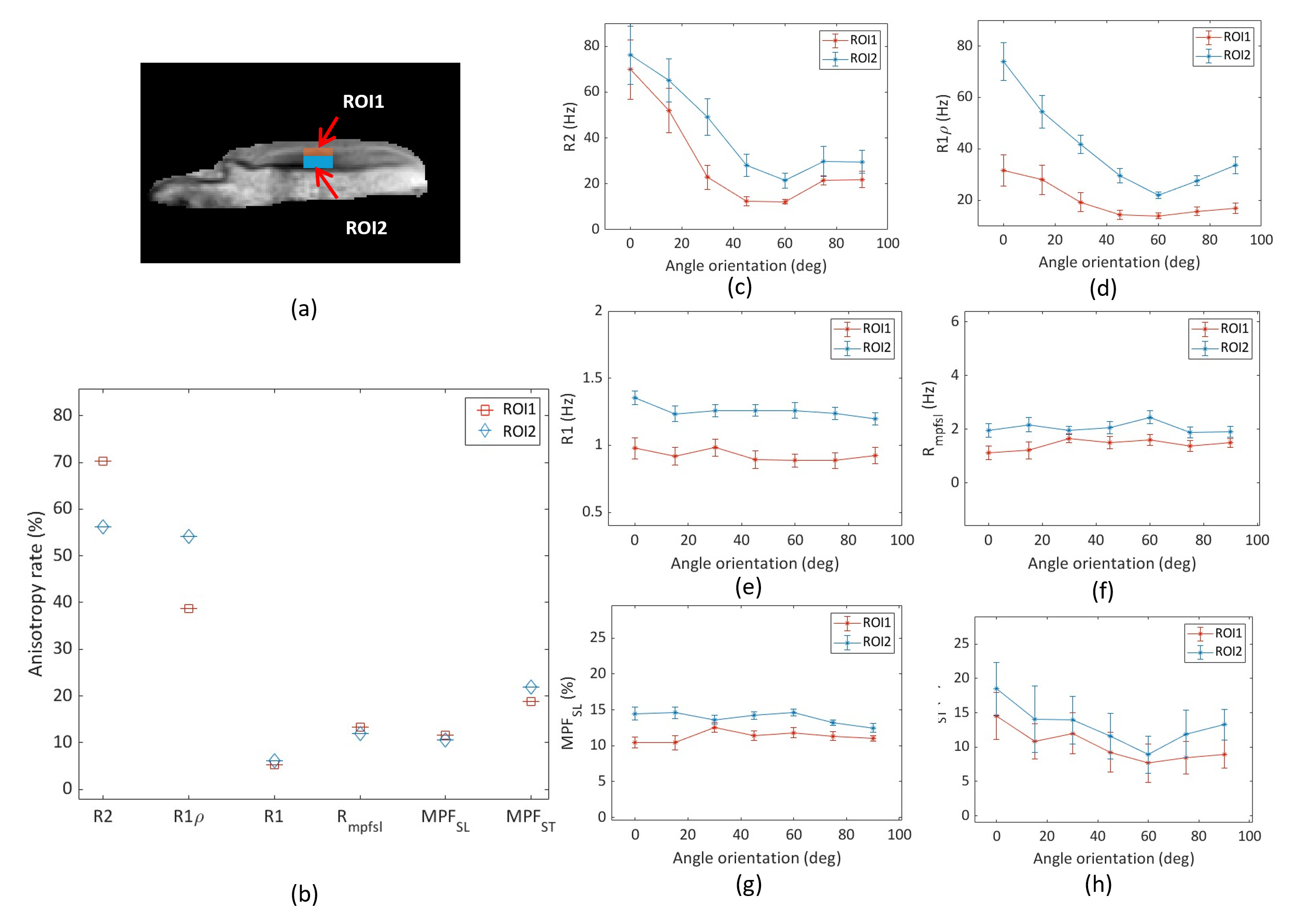


Figure. S4. (a) Illustration of two ROIs in knee specimen S3. (b) Anisotropy rate of relaxation time maps ($R_{2}$, $R_{\text{1ρ}}$, $R_{1}$, and $R_{mpfsl}$) and MPF maps ($MPF_{SL}$ and $MPF_{ST}$) in ROI1 and ROI2. (c-h) Mean ± standard deviation of relaxation rates in ROI1 and ROI2, as shown in (a), were calculated at different angle orientations (c) $R_{2}$, (d) $R_{\text{1ρ}}$, (e) $R_{1}$, (f) $R_{mpfsl}$, (g) $MPF_{SL}$, and (h) $MPF_{ST}$.


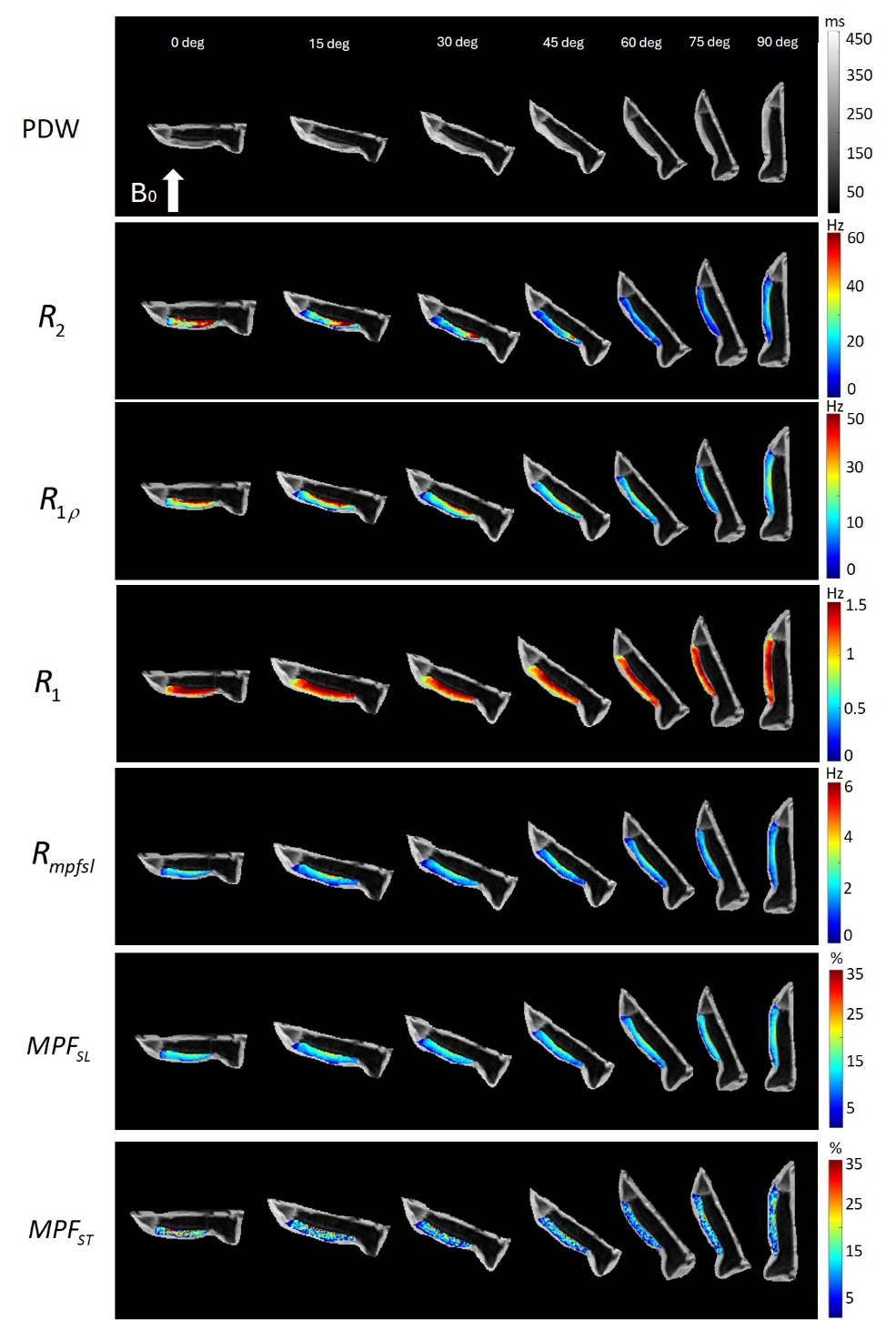


Figure. S5. Maps of relaxation rates and MPF of knee specimen S4 at different orientations. Top to the bottom: maps of $R_{2}$, on-resonance $R_{\text{1ρ}}$ at FSL 500 Hz, $R_{1}$, $R_{mpfsl}$, $MPF_{SL}$, and $MPF_{ST}$. Left to right: maps at orientations of 0°, 15°, 30°, 45°, 60°, 75°, and 90° with respect to *B*_0_. PDW images are included as anatomical reference images.


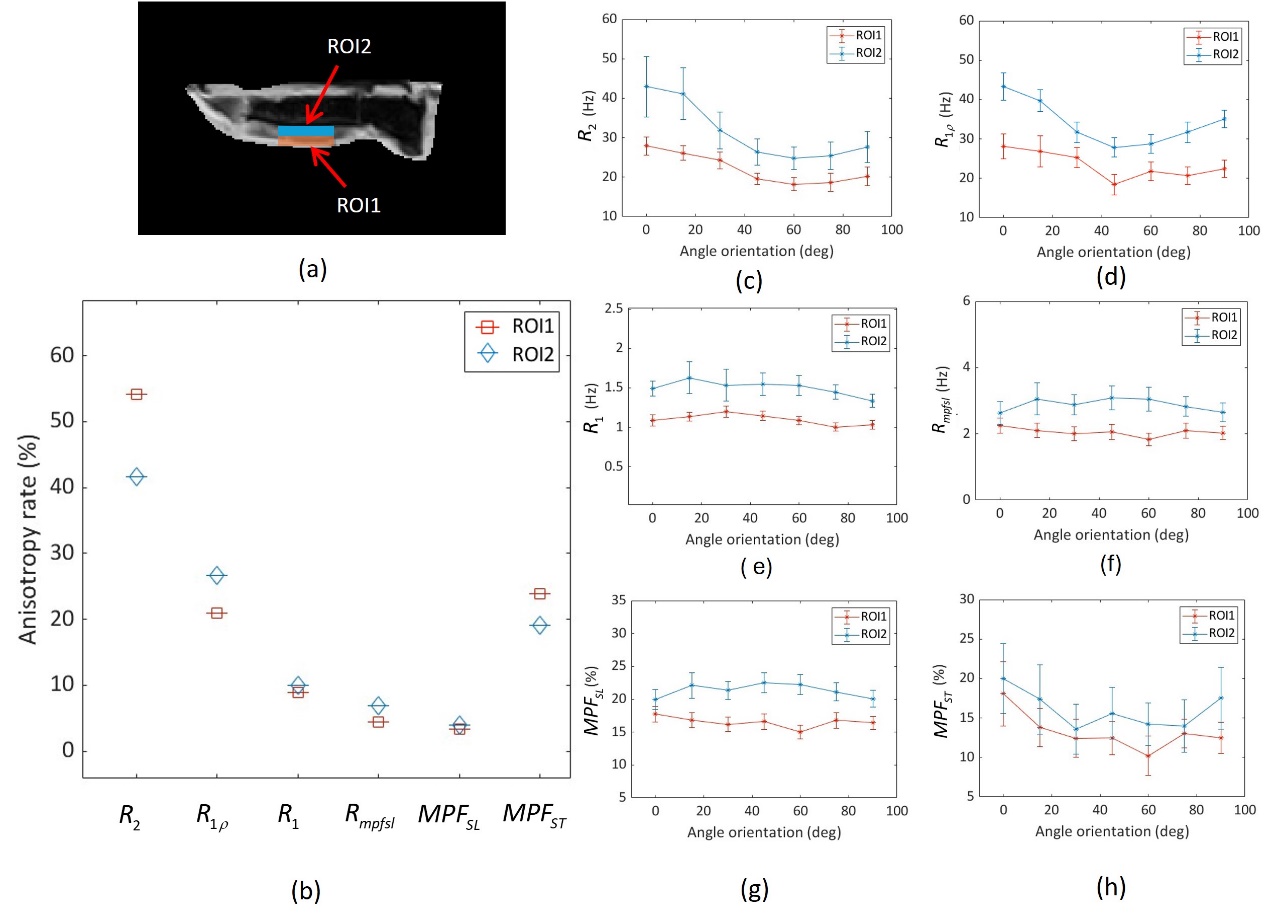


Figure. S6. (a) Illustration of two ROIs in knee specimen S4. (b) Anisotropy rate of relaxation time maps ($R_{2}$, $R_{\text{1ρ}}$, $R_{1}$, and $R_{mpfsl}$) and MPF maps ($MPF_{SL}$ and $MPF_{ST}$) in ROI1 and ROI2. (c-h) Mean ± standard deviation of relaxation rates in ROI1 and ROI2, as shown in (a), were calculated at different angle orientations (c) $R_{2}$, (d) $R_{\text{1ρ}}$, (e) $R_{1}$, (f) $R_{mpfsl}$, (g) $MPF_{SL}$, and (h) $MPF_{ST}$.


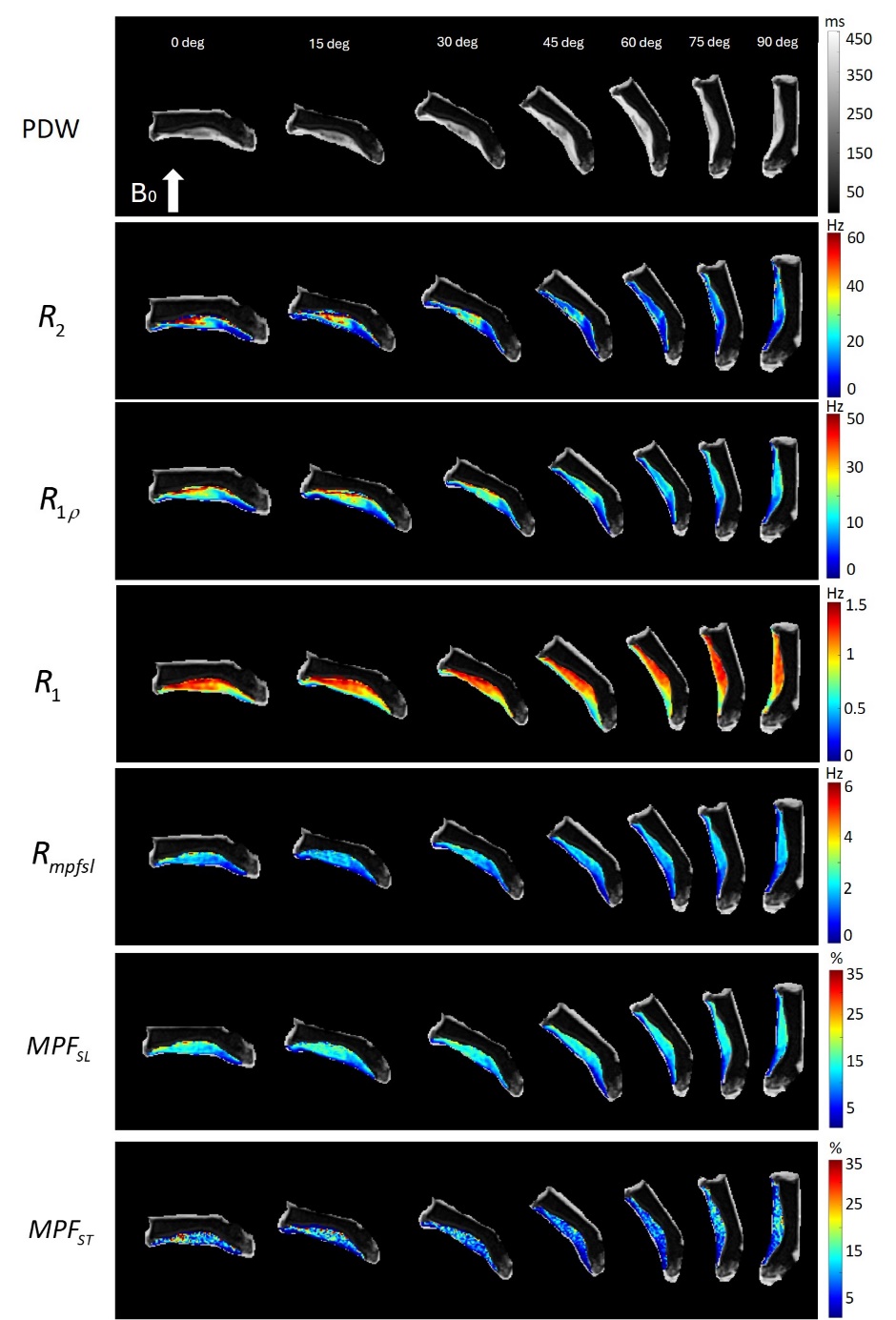


Figure. S7. Maps of relaxation rates and MPF of knee specimen S5 at different orientations. Top to the bottom: maps of $R_{2}$, on-resonance $R_{\text{1ρ}}$ at FSL 500 Hz, $R_{1}$, $R_{mpfsl}$, $MPF_{SL}$, and $MPF_{ST}$. Left to right: maps at orientations of 0°, 15°, 30°, 45°, 60°, 75°, and 90° with respect to *B*_0_. PDW images are included as anatomical reference images.


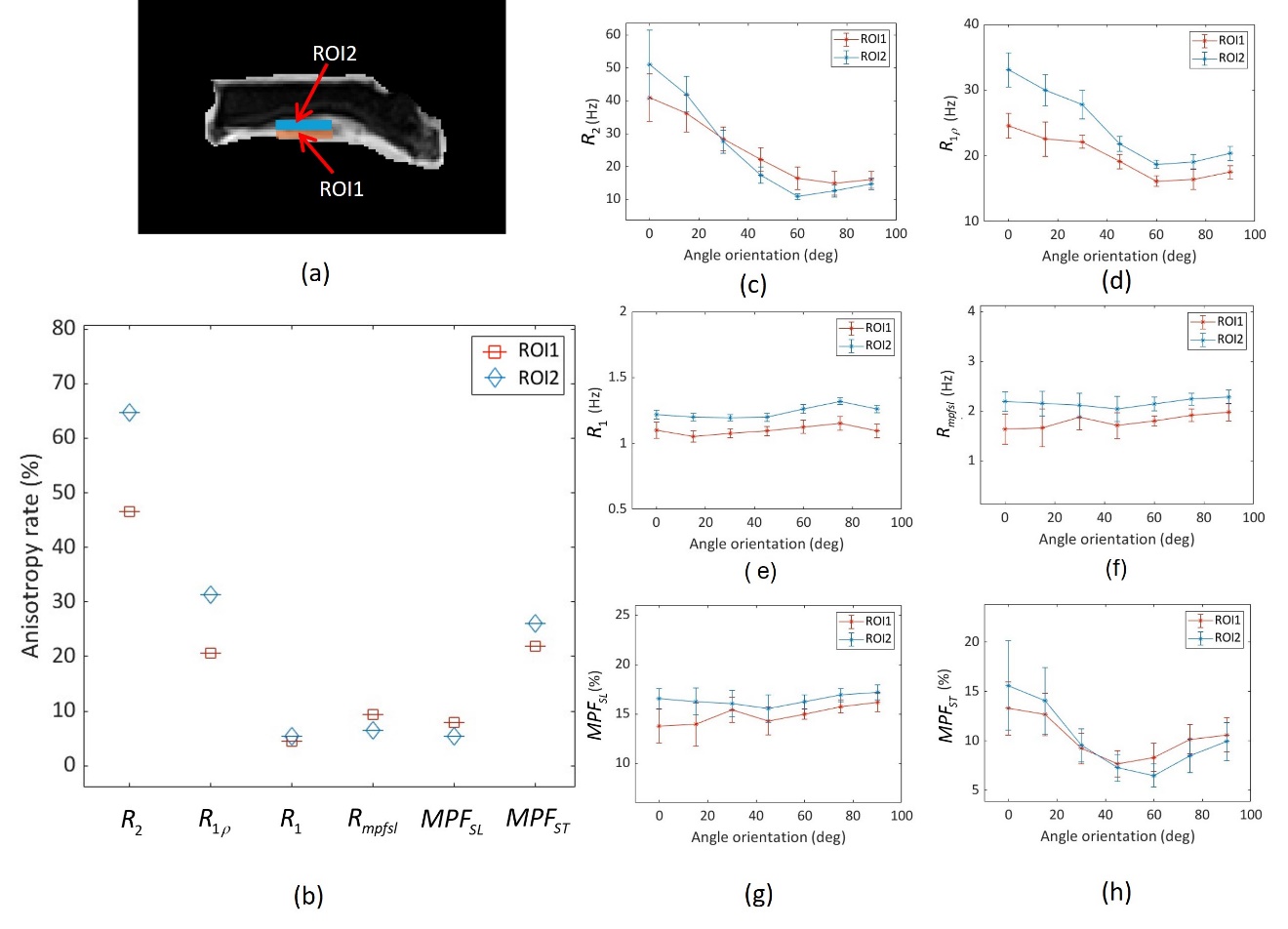


Figure. S8. (a) Illustration of two ROIs in knee specimen S5. (b) Anisotropy rate of relaxation time maps ($R_{2}$, $R_{\text{1ρ}}$, $R_{1}$, and $R_{mpfsl}$) and MPF maps ($MPF_{SL}$ and $MPF_{ST}$) in ROI1 and ROI2. (c-h) Mean ± standard deviation of relaxation rates in ROI1 and ROI2, as shown in (a), were calculated at different angle orientations (c) $R_{2}$, (d) $R_{\text{1ρ}}$, (e) $R_{1}$, (f) $R_{mpfsl}$, (g) $MPF_{SL}$, and (h) $MPF_{ST}$.


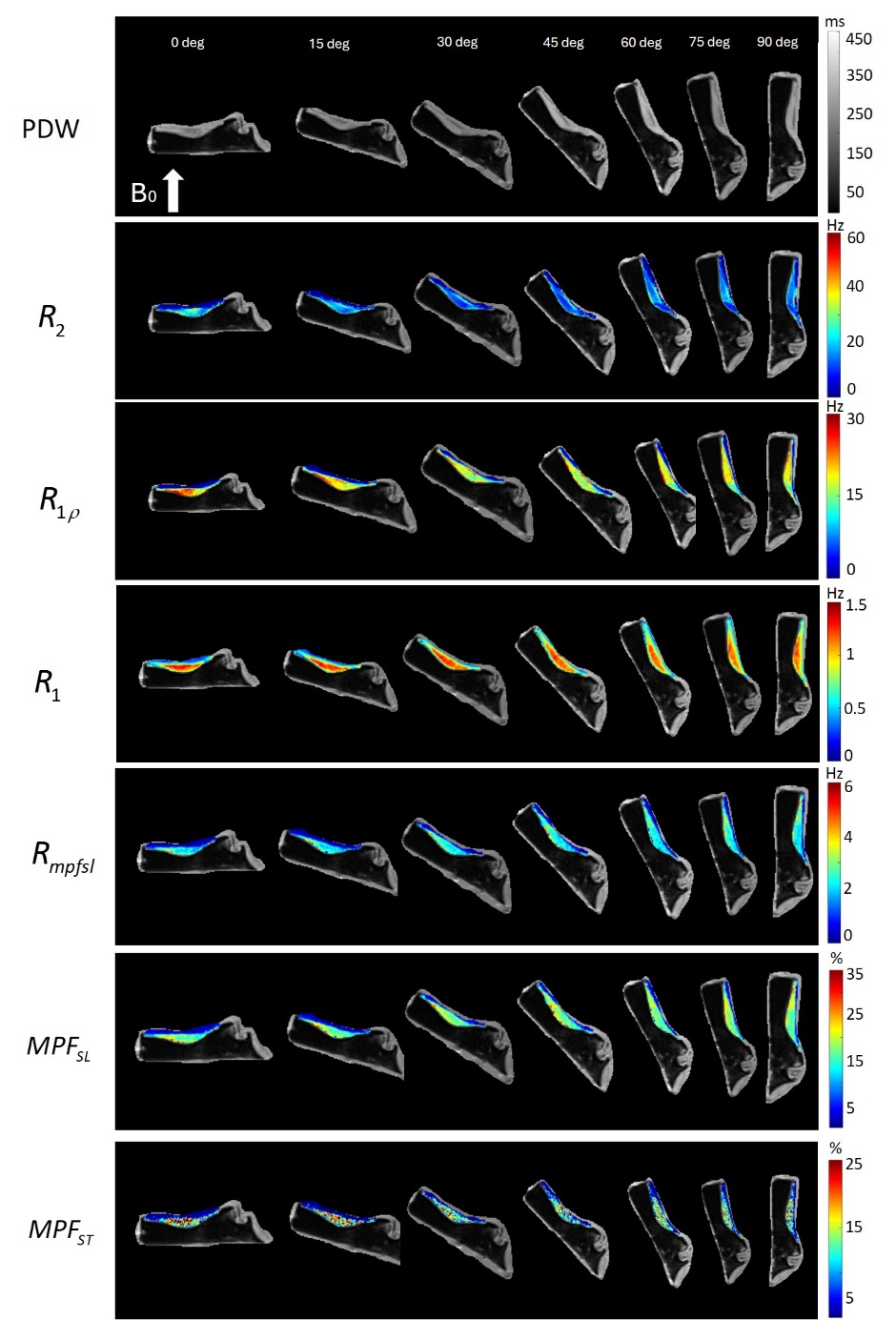


Figure. S9. Maps of relaxation rates and MPF of knee specimen S6 at different orientations. Top to the bottom: maps of $R_{2}$, on-resonance $R_{\text{1ρ}}$ at FSL 500 Hz, $R_{1}$, $R_{mpfsl}$, $MPF_{SL}$, and $MPF_{ST}$. Left to right: maps at orientations of 0°, 15°, 30°, 45°, 60°, 75°, and 90° with respect to *B*_0_. PDW images are included as anatomical reference images.


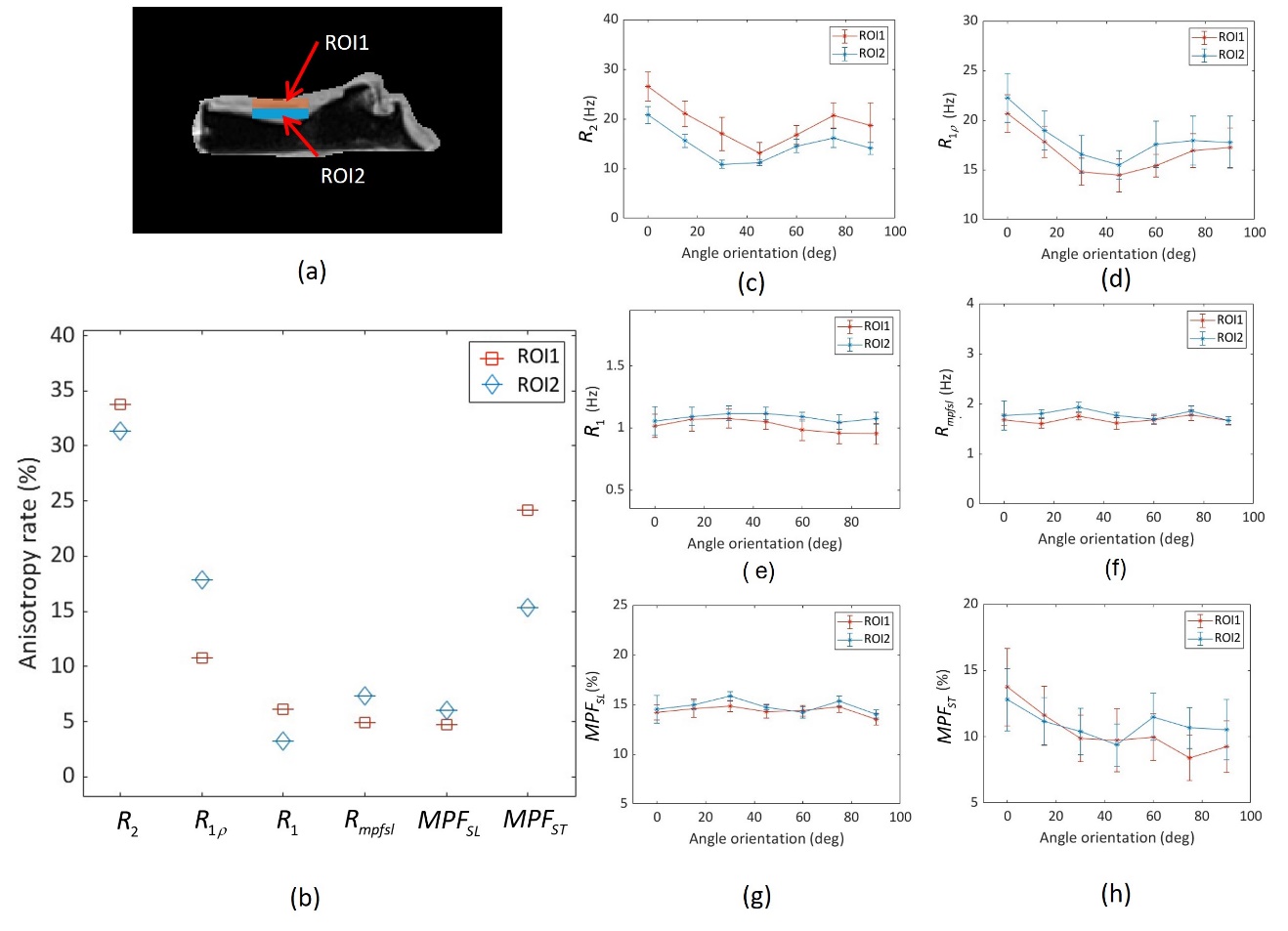


Figure. S10. (a) Illustration of two ROIs in knee specimen S6. (b) Anisotropy rate of relaxation time maps ($R_{2}$, $R_{\text{1ρ}}$, $R_{1}$, and $R_{mpfsl}$) and MPF maps ($MPF_{SL}$ and $MPF_{ST}$) in ROI1 and ROI2. (c-h) Mean ± standard deviation of relaxation rates in ROI1 and ROI2, as shown in (a), were calculated at different angle orientations (c) $R_{2}$, (d) $R_{\text{1ρ}}$, (e) $R_{1}$, (f) $R_{mpfsl}$, (g) $MPF_{SL}$, and (h) $MPF_{ST}$.


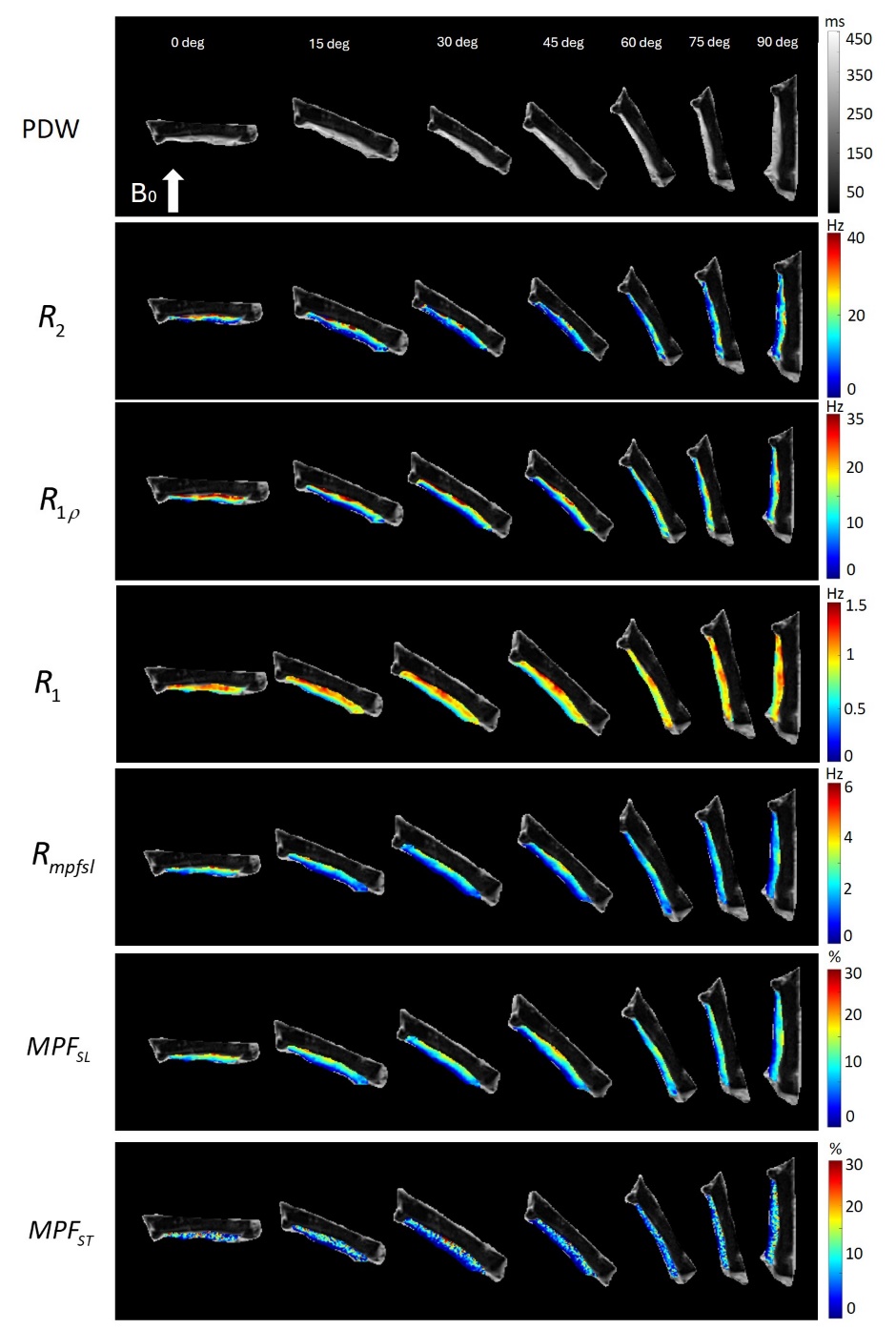


Figure. S11. Maps of relaxation rates and MPF of knee specimen S7 at different orientations. Top to the bottom: maps of $R_{2}$, on-resonance $R_{\text{1ρ}}$ at FSL 500 Hz, $R_{1}$, $R_{mpfsl}$, $MPF_{SL}$, and $MPF_{ST}$. Left to right: maps at orientations of 0°, 15°, 30°, 45°, 60°, 75°, and 90° with respect to *B*_0_. PDW images are included as anatomical reference images.


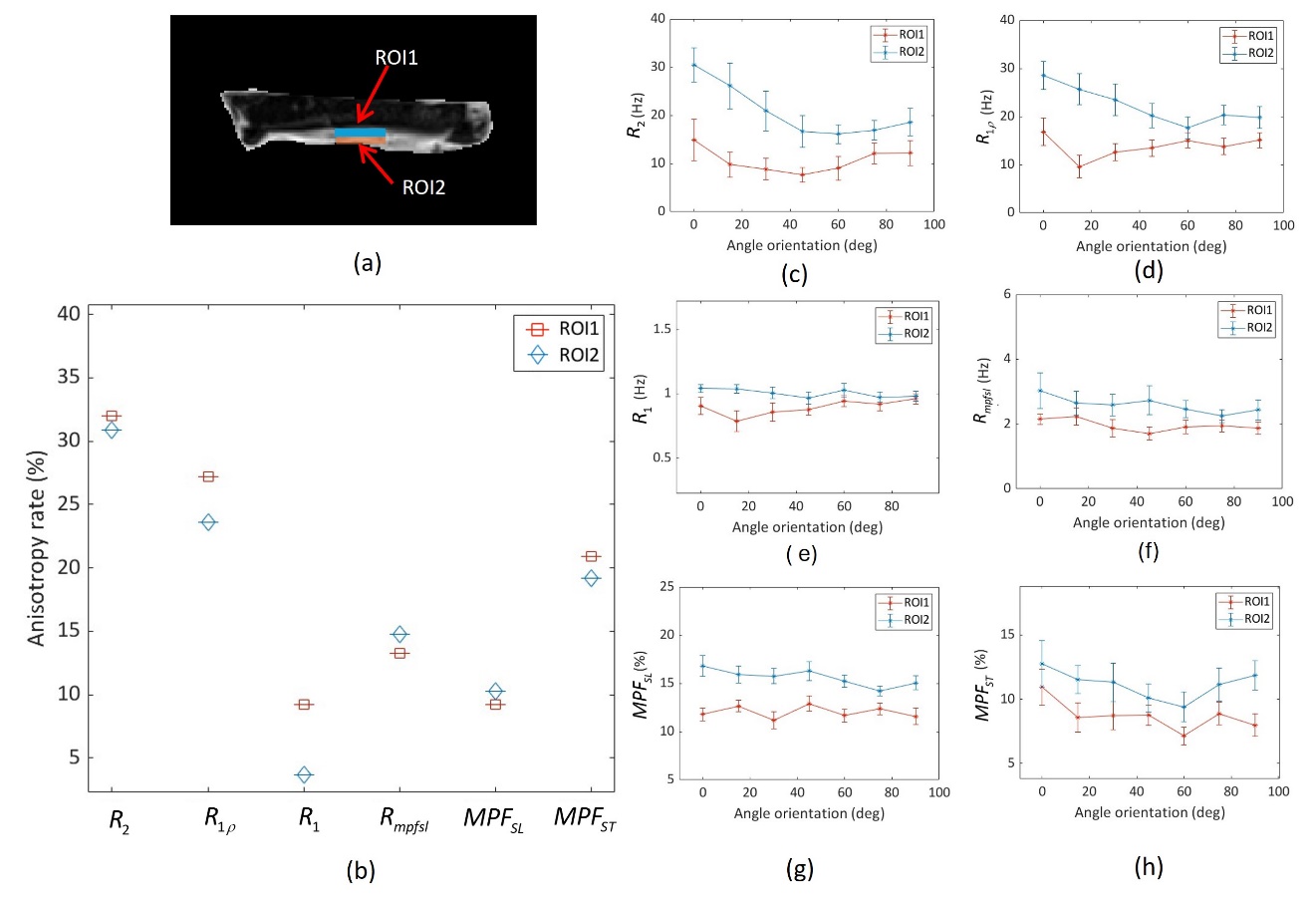


Figure. S12. (a) Illustration of two ROIs in knee specimen S7. (b) Anisotropy rate of relaxation time maps ($R_{2}$, $R_{\text{1ρ}}$, $R_{1}$, and $R_{mpfsl}$) and MPF maps ($MPF_{SL}$ and $MPF_{ST}$) in ROI1 and ROI2. (c-h) Mean ± standard deviation of relaxation rates in ROI1 and ROI2, as shown in (a), were calculated at different angle orientations (c) $R_{2}$, (d) $R_{\text{1ρ}}$, (e) $R_{1}$, (f) $R_{mpfsl}$, (g) $MPF_{SL}$, and (h) $MPF_{ST}$.
